# Theoretical and Simulation-Based Investigation of the Relationship between Sequencing Effort, Microbial Community Richness, and Diversity in Binning Metagenome-Assembled Genomes

**DOI:** 10.1128/mSystems.00384-19

**Published:** 2019-09-17

**Authors:** Taylor M. Royalty, Andrew D. Steen

**Affiliations:** a Department of Earth and Planetary Sciences, University of Tennessee, Knoxville, Tennessee, USA; b Department of Microbiology, University of Tennessee, Knoxville, Tennessee, USA; Pacific Northwest National Laboratory

**Keywords:** DNA sequencing, MAG, ecology, mathematical modeling, metagenomics, microbial communities

## Abstract

We applied theoretical and simulation-based approaches to characterize how microbial community structure influences the amount of sequencing effort to reconstruct metagenomes that are assembled from short-read sequences. First, a coupon collector equation was proposed as an analytical model for predicting sequencing effort as a function of microbial community structure. Characterization was performed by varying community structure properties such as richness, evenness, and genome size. Simulations demonstrated that while community richness and evenness influenced the sequencing effort required to sequence a community metagenome to exhaustion, the effort necessary to sequence an individual genome to a target fraction of exhaustion depended only on the relative abundance of the genome and its genome size. A second analysis evaluated the quantity, completion, and contamination of metagenome-assembled genomes (MAGs) as a function of sequencing effort on four preexisting sequence read data sets from different environments. These data sets were subsampled to various degrees of completeness to simulate the effect of sequencing effort on MAG retrieval. Modeling suggested that sequencing efforts beyond what is typical in published experiments (1 to 10 Gbp) would generate diminishing returns in terms of MAG binning. A software tool, Genome Relative Abundance to Sequencing Effort (GRASE), was created to assist investigators to further explore this relationship. Reevaluation of the relationship between sequencing effort and binning success in the context of genome relative abundance, as opposed to base pairs, provides a constraint on sequencing experiments based on the relative abundance of microbes in an environment rather than arbitrary levels of sequencing effort.

**IMPORTANCE** Short-read sequencing with Illumina sequencing technology provides an accurate, high-throughput method for characterizing the metabolic potential of microbial communities. Short-read sequences can be assembled and binned into metagenome-assembled genomes, thus shedding light on the function of microbial ecosystems that are important for health, agriculture, and Earth system processes. The work presented here provides an analytical framework for selecting sequencing effort as a function of genome relative abundance. As such, experimental goals in metagenome-assembled genome creation projects can select sequencing effort based on the rarest target genome as a constrained threshold. We hope that the results presented here, as well as GRASE, will be valuable to researchers planning sequencing experiments.

**Author Video**: An author video summary of this article is available.

## Abstract (precis)

Short-read sequencing with Illumina sequencing technology provides an accurate, high-throughput method for characterizing the metabolic potential of microbial communities. Short-read sequences can be assembled and binned into metagenome-assembled genomes, thus shedding light on the function of microbial ecosystems that are important for health, agriculture, and Earth system processes. The work presented here provides an analytical framework for selecting sequencing effort as a function of genome relative abundance. As such, experimental goals in metagenome-assembled genome creation projects can select sequencing effort based on the rarest target genome as a constrained threshold. We hope that the results presented here, as well as GRASE, will be valuable to researchers planning sequencing experiments.

## INTRODUCTION

The reconstruction of high-accuracy short-read sequences into metagenome-assembled genomes (MAGs) is a powerful approach to characterize microbial metabolisms within complex communities (1). The recent creation of ∼8,000 MAGs from largely uncultured organisms across the tree of life (2), the spatial characterization of microbial metabolisms and ecology across Earth’s oceans (3), and the characterization of the potential impact that fermentation-based microbial metabolisms have on biogeochemical cycling in subsurface sediment environments (4) provide a few examples of how MAGs have helped to elucidate the relationships between microbial ecology, microbial metabolisms, and biogeochemistry.

**FIG 1:**
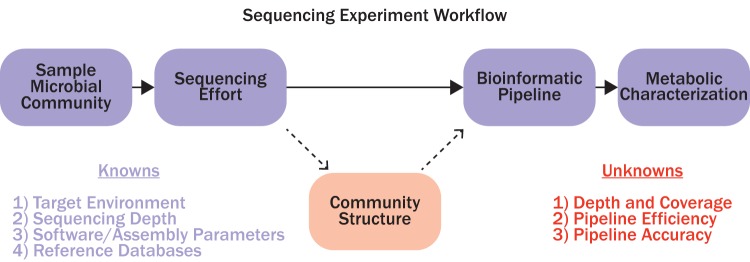
A flow diagram illustrating the workflow for sequencing experiments.

Sampling environmental microbial DNA involves selecting a target environment, sequencing effort, bioinformatic pipeline software and parameters, metabolism characterization software (i.e., for gene identification and similarity searches), and databases (Fig. 1). At present, there is little information to guide how much sequencing is appropriate to achieve scientific goals in such experiments (5). This gap in knowledge is partly attributed to the unknown structure of target microbial communities. A further challenge is that the accuracy and efficiency of bioinformatic pipelines are often difficult to characterize, and thus obscure the relationship between sequencing effort and MAG retrieval. Recent estimates compiled by Quince et al. (5) suggest that typical metagenomic shotgun sequencing experiments usually sequence between 1 Gbp and 10 Gbp. Researchers require more precise guidance to select an appropriate shotgun sequencing effort in order to maximize information and minimize cost for their specific experimental question.

Illumina sequencing technology is currently the most popular platform to generate metagenomic shotgun sequences (5). Previous investigators established theoretical relationships between contig formation rate (6) and single genome coverage (7) as a function of short-read sequencing effort. On the community level, heuristic approaches have been proposed for evaluating community-level coverage in respect to increases in sequencing effort. For example, it has been proposed to utilize short-read redundancies as a function of sequencing effort to estimate community-level coverage (8). Without *a priori* knowledge of the microbial community structure, practical application of these methods to estimate MAG retrieval as a function of sequencing effort is hindered.

Here, we present two distinct analyses which constrain the relationship between the quantity of Illumina metagenomic shotgun sequences and the community-level sequence coverage. First, we applied a theoretical model and numerical simulations to estimate the minimum sequencing effort needed to sequence a metagenome to a target fraction of exhaustion. Our theoretical model is unique compared to previous models (6, 7) in that we characterize sequencing effort in the context of community structure and consider all sequenceable combinations of *k*-mers in a metagenome. Second, we performed *in silico* experiments to simulate the effect of sequencing effort on retrieved MAGs for Illumina sequence data sets. Coupling results from the two analyses provides a framework for investigators to define sequencing experiments in the context of selecting a rarity and fraction of exhaustion for a desired target genome when sequencing a community. The patterns presented here can be used to guide sequencing effort decisions in future sequencing projects when MAG reconstruction is a primary goal.

## RESULTS

### Theoretical and numerical sequencing effort simulations.

**FIG 2:**
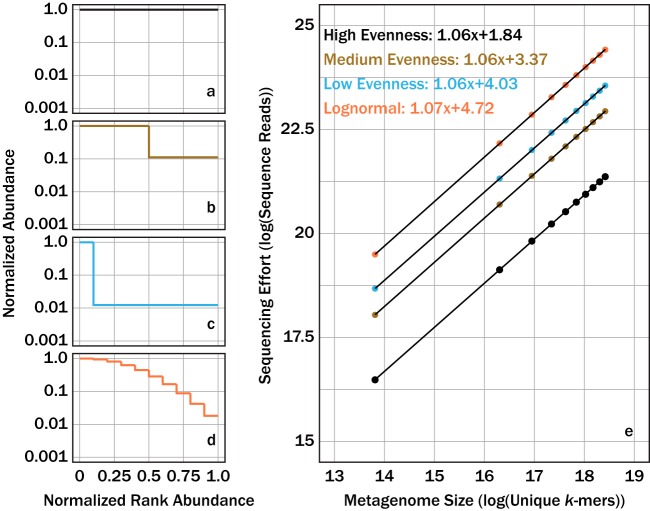
Sequencing effort, with units of log of sequence reads, required to fully sequence four different community structures, one with relatively high community evenness (a), relatively moderate community evenness (b), relatively low community evenness (c), and one with a lognormal community structure (d), were predicted using linear regressions (e) and the log of metagenome size (total possible *k*-mers) as a predictor.

Using equation 6, we calculated the number of sequence reads required to sequence four hypothetical microbial communities to exhaustion: a perfectly even, moderately uneven, highly uneven, and a lognormally distributed structured community (Fig. 2a to d). Here, we define sequencing a community to exhaustion as sequencing all possible combinations of DNA *k*-mers within a genome. Note that, to simplify the model, this model treats identical *k*-mers (i.e., same DNA sequence) at different locations in the genome as mathematically different *k*-mers. The expected number of sequence reads to fully sequence the hypothetical communities was linear after log-transforming both expected sequences and metagenome size (unit of unique *k*-mers), suggesting a power-law relationship between metagenome size and the number of sequence reads required to sequence the metagenome to exhaustion (Fig. 2e). For all community structures, the slope of the relationship between log-transformed sequencing effort (sequenced reads) and metagenome size (unique *k*-mers) was within 1% of 1.06. The structure of the population strongly influenced the number of reads required such that more-even community structures required far fewer reads than less-even structures.

**FIG 3:**
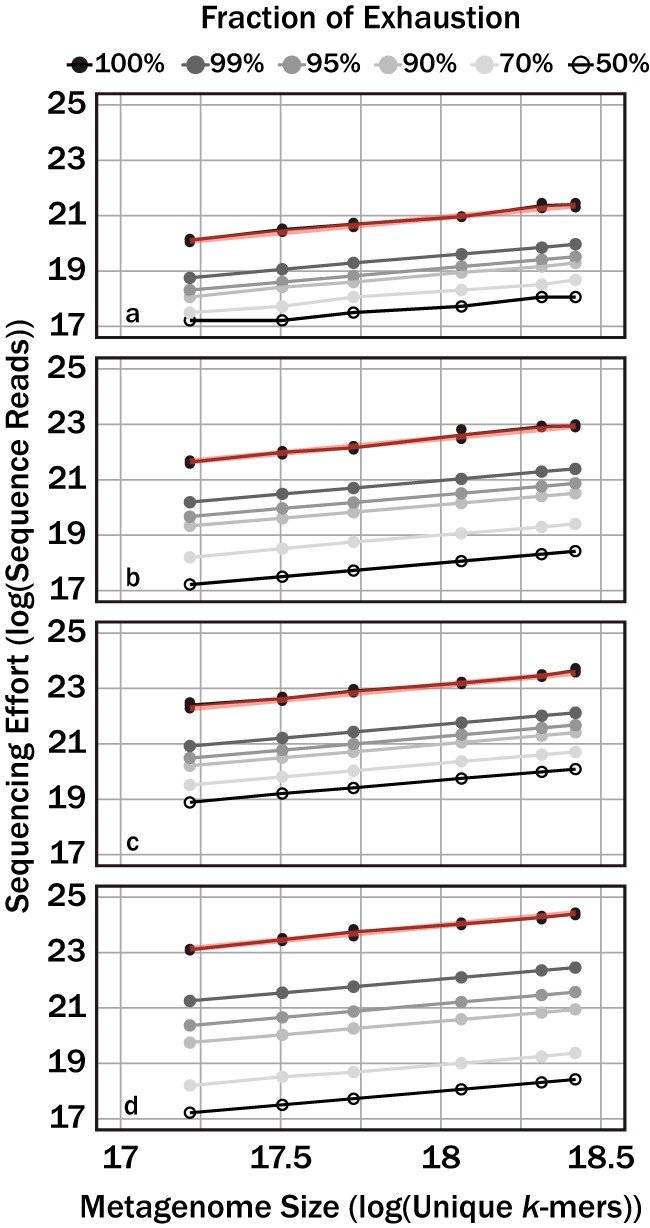
Sequencing effort, with units of log sequence reads, necessary to reach variable target sequencing depths (colors) for four different community structures, one with relatively high community evenness (a), relatively moderate community evenness (b), relatively low community evenness (c), and one with a lognormal community structure (d). Red translucent lines correspond with linear regression curves for the respective community in Fig. 2e.

A limitation from using equation 6 for modeling sequencing effort is that it estimates only the sequencing effort for sequencing a metagenome to exhaustion. We circumnavigated this limitation by applying a numerical simulation to estimate the sequencing effort necessary to sequence a metagenome to a fraction of exhaustion for the same community structures analyzed earlier. The numerical simulation was validated by comparing the sequencing effort predicted from the numerical simulation and those from equation 6 when sequencing a metagenome to exhaustion (Fig. 3). Again, the relationship between metagenome size and sequencing effort fit well to a power law. This observation was independent of the selected target fraction of exhaustion. Nonetheless, an increase in target fraction of exhaustion resulted in uniform increases in sequencing effort (log units) when the community structure was fixed; however, the rate of this increase varied with community structure evenness.

**FIG 4:**
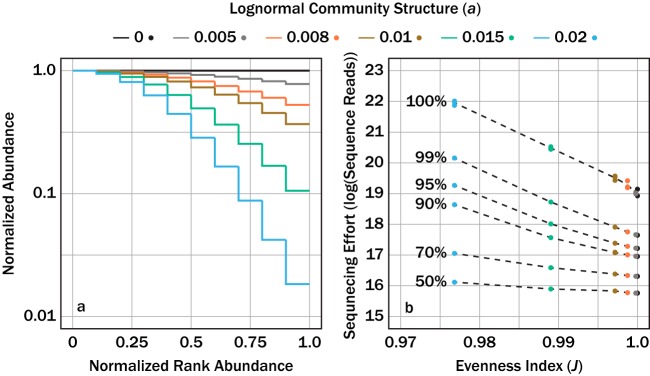
Numerical sequencing simulations applied to six hypothetical communities with different lognormal distributions that were defined by the parameter *a* from equation 7 (a). The sequencing effort, with units of log of sequence reads, necessary to sequence a target fraction of exhaustion (dashed contours) as a function of the Pielou evenness index *J* for a given lognormal community structure (b).

We were interested in quantitatively relating community evenness to sequencing effort. These communities ranged from perfectly even (*a *=* *0, equation 8) to more uneven (*a *=* *0.02, Fig. 4a). Evenness was quantified using the Pielou evenness index, which expresses Shannon diversity relative to the diversity of a perfectly even community (9). The sequencing effort required to characterize genomes depended strongly on both the evenness and the target fraction of exhaustion (Fig. 4b). Again, less-even communities required more sequence reads than more-even communities did. The strength of this relationship also depended on the target fraction of completion. A community with a Pielou evenness of 0.97 required 3 orders of magnitude more sequencing effort to sequence a metagenome to a target fraction of exhaustion than a perfectly even community, while the same community required only about 42% more reads to sequence 50% of the metagenome.

**FIG 5:**
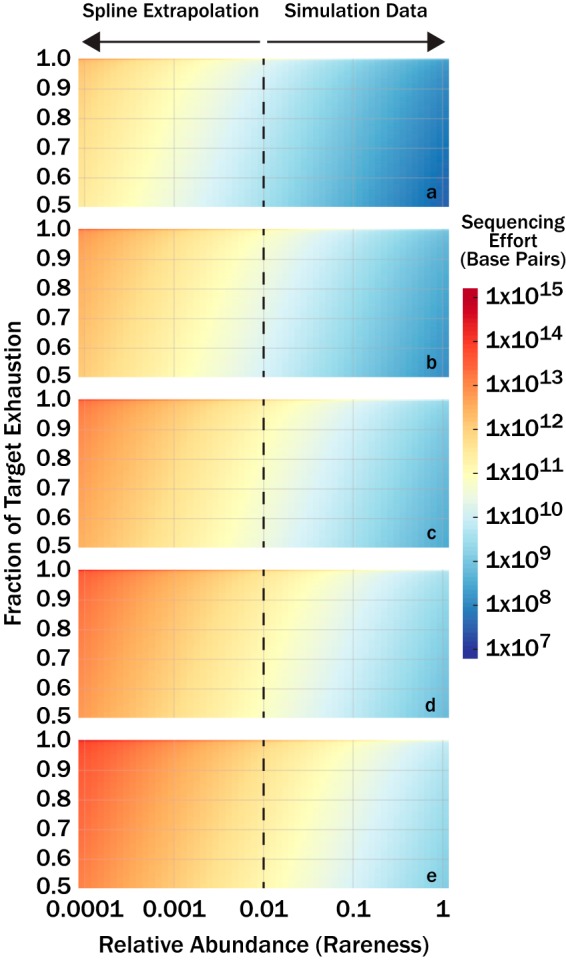
Numerical sequencing simulations show the number of bases (color bar) required to sequence a target fraction of a genome as a function of that genome’s relative abundance in the community metagenome. Genomes evaluated were 0.5 × 10^6^ (a), 2 × 10^6^ (b), 5 × 10^6^ (c), 10 × 10^6^ (d), and 20 × 10^6^ (e) base pairs long.

The inherent limitation to the theoretical and numerical constraints presented above is that community structure is not known *a priori*. Nonetheless, a simple line of rationalization can be applied to circumnavigate this issue for practical applications of our model. Equation 6 constrains expected sequencing effort based on the proportion of the population under consideration. That is, we can limit the model to consider only DNA *k*-mers associated with a set of genomes that represent a certain fraction of the community. Here, we limit the population such that our model predicts the expected sequencing effort of the rarest genome that we wish to sequence. Inherently, any member more abundant than the selected rarest genome will also be sequenced to the minimum coverage and depth of the selected rarest genome. To determine the rareness of a given genome within a metagenome, the total number of unique *k*-mers within a genome (equation 2) is scaled by the true abundance of the microbe and divided by the size of the metagenome (equation 7). Thus, the proper application of this rationale requires a desired target fraction of exhaustion, an assumed genome size, and the relative abundance of the rarest genome to sequence. Numerical simulations, like those described earlier, were performed to determine the sequencing effort necessary to achieve a target fraction of exhaustion. The difference here is that these simulations analyzed the sequencing effort necessary to sequence a genome of a certain rareness to a target fraction of exhaustion, whereas the simulations above analyze the effort necessary to sequence the entire community. A generalized additive model (GAM) was built from simulation outputs to extrapolate sequencing effort required for relative abundances of less than 1% as simulations with lower abundances became computationally too intensive (GAM) (Fig. 5). The GAM shows expected sequencing effort required for microbial genome sizes of 0.5, 2, 5_,_ 10, and 20 Mbp, target genome completeness fractions from 0.5 to 1.0, and genome relative abundances from 1 to 0.0001. The smooth dimensions for target fraction, genome size, and fraction of the metagenome community were 50, 6, and 29, respectively. To normalize for different sequence read length, sequence reads were converted to bases and ranged from 1 × 10^7^ to 1 × 10^15^ total bases. More bases were required to sequence microorganisms (i) when the genome was relatively rarer in the community, (ii) to achieve better coverage of the genome, and (iii) when average genome sizes were larger.

### Rarefying MAG binning as a function of sequencing effort.

**FIG 6:**
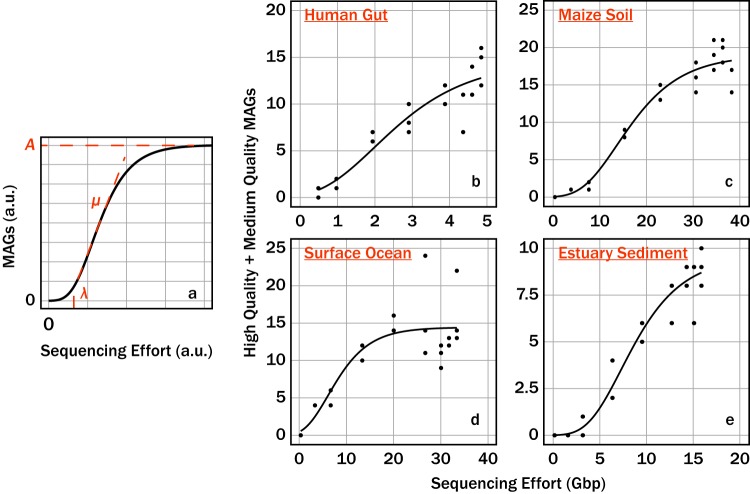
(a) The influence that the parameters *A*, μ, and λ had on the Gompertz equation. The sequencing effort and MAGs are shown in arbitrary units (a.u.). The property of the Gompertz equation that each parameter influences is colored red. The sum of medium-quality and high-quality MAGs (quality MAGs) as a function of sequencing effort for the gut (b), soil (c), surface ocean (d), and sediment (e) communities. The solid lines in panels b to e correspond to nonlinear least-squares fits of the Gompertz equation to the respective environmental data set. Note the different scales for the axes in panels b to e.

We rarefied four sequence read data sets to nine different fractions of the complete sequence data sets in triplicate. The rarefied data sets were then assembled and binned for a total of 108 analyzed metagenomes. Raw MAG data are provided in Data Set S1 in the supplemental material. The sum of medium- and high-quality MAGs as a function of high-quality bases empirically fit the Gompertz equation (equation 12; Fig. 6b to e; parameters in Table 1; Data Set S1). The sum of medium- and high-quality MAGs (henceforth referred to as quality MAGs for brevity) reduces sensitivity to whether bioinformatic pipelines tend to lump contigs into fewer, more-complete MAGs versus split them into more, less-complete MAGs. This was important for our analysis due to the large number of metagenomes (*n *=* *108) which required assembly and an unsupervised binning algorithm. The large number of metagenomes made manual bin curation impractical during rarefication simulations.

**TABLE 1:** Estimates of fit coefficients for the Gompertz equation (equation 12) for the sum of high-quality and medium-quality MAGs as a function of sequencing depth in published data sets from ocean surface water, estuarine sediment, maize soil, and the human gut^*a*^

Environment	*A* (SE)	μ (SE)	λ (SE)	Yield
Ocean surface water	14.4 (1.0)	1.1 (0.4)	1.2 (2.1)	1.00
Estuary sediment	9.6 (0.9)	1.0 (0.2)	3.7 (0.7)	0.91
Maize soil	19.0 (1.1)	0.9 (0.1)	6.1 (1.5)	0.96
Human gut	14.6 (2.2)	3.8 (0.7)	0.6 (0.3)	0.88

a *P* values for all coefficients were <<0.05 except for gut and ocean surface water λ values. Yield is defined in equation 13 as the number of medium- and high-quality MAGs relative to the number predicted at infinite sequencing depth.

10.1128/mSystems.00384-19.2 DATA SET S1 Raw MAG data generated from the assembly pipeline. Download Data Set S1, CSV file, 6.7 MB. Copyright © 2019 Royalty and Steen. 2019 Royalty and Steen This content is distributed under the terms of the Creative Commons Attribution 4.0 International license.

For each environment, the quality MAGs as a function of simulated sequencing effort followed a sigmoidal relationship. In order to make the parameters of the fit intuitive to interpret, we fit the data to the Gompertz equation as rewritten by Zwietering et al. (10) (equation 12). Here, *A*, *μ*, and λ correspond to the maximum quality MAGs assembled with the pipeline, the maximum rate which the quality MAGs form with more sequencing, and the “lag bases,” or the bases which must be sequenced before a sufficient number of sequence reads exist to generate overlap and form contigs (6). The predicted maximum quality MAGs varied from ∼9.6 in the estuary sediment community to ∼19 in the maize soil community. The predicted maximum rate that the quality MAGs increased varied from ∼0.9 to ∼3.8 MAGs Gbp^−1^. Last, the minimum threshold of sequencing necessary prior to seeing quality MAGs varied from ∼0.6 to ∼6.1 Gbp. The *Tara Oceans* data set, where the quality decreased at a sequencing effort of >20 Gbp, was an exception. For the estuary, maize, and human gut data sets, the quality MAGs yield began to asymptote with increasing sequencing efforts. The *Tara Oceans* data set followed a similar pattern at <25 Gbp. However, when the number of sequenced bases was >25 Gbp, the quality MAGs decreased and became insensitive to sequencing effort.

### Constraining MAG rarefaction analyses to community structure.

**FIG 7:**
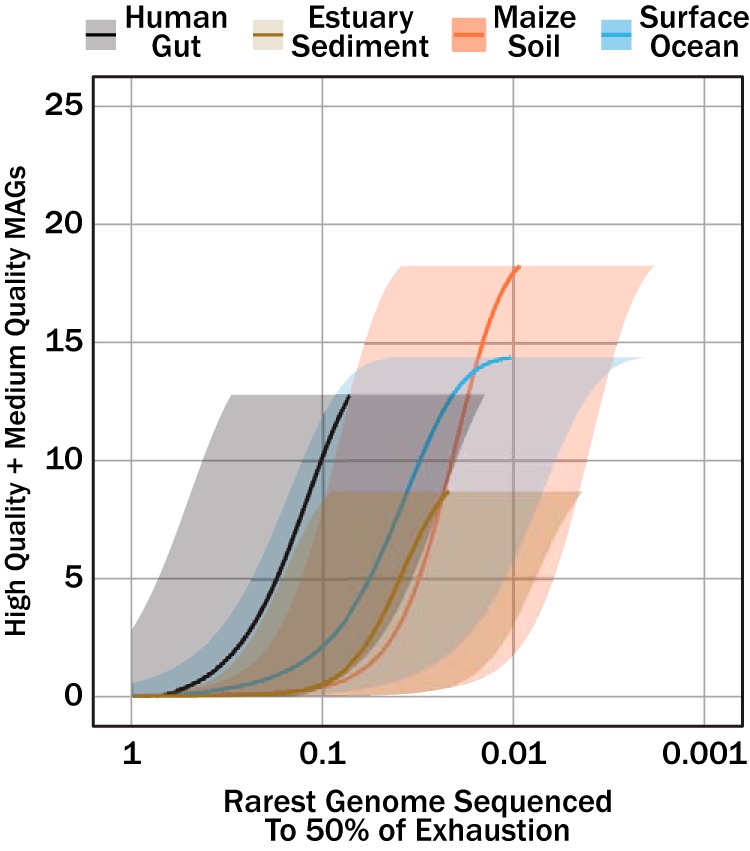
Quality MAGs (medium and high quality) as a function of the rarest genome sequenced to 50% exhaustion for human gut, maize soil, estuarian sediment, and surface ocean sequence data sets. Sequencing effort was converted to genome relative abundance sequenced to target fraction using the GAM presented in Fig. 5 The translucent shaded areas correspond to uncertainty from the target genome size (1 Mbp, or the lower bound, to 20 Mbp, or the upper bound), while the solid lines correspond to genome sizes of 5 Mbp.

Using the relationship shown in Fig. 5, we can convert sequencing effort to an abundance (rareness) cutoff if we assume a genome size. Genome sizes for genomes in RefSeq v92 (11) have 25th, 50th, and 75th quantiles of 2.73 Mbp, 4.30 Mbp, and 5.14 Mbp, respectively, and provide reasonable constraints for assumptions of genome size. Figure 7 shows quality MAGs retrieved for a genome of a given level of abundance that is sequenced to a target fraction of completeness for the human gut, maize soil, estuarine sediment, and surface ocean microbiomes analyzed earlier. Correlation coefficients for the regressions used to relate log-transformed sequencing effort (in base pairs) to genome relative abundance were *R *=* *1 for all three genome sizes evaluated (1 Mbp, 5 Mbp, and 20 Mbp). Evaluation of 1 Mbp and 20 Mbp define the range of uncertainty in predicting quality MAGs as the true size of genomes is unknown. Unlike the asymptotic response of quality MAGs to sequencing effort (Fig. 6b to e), quality MAGs increase exponentially as the abundance cutoff decreases (note that the abundance cutoff is on a log scale). The target genome completeness fraction was held at a constant of 0.5 for all regressions, and the sensitivity of quality MAGs will change with respect to the genome abundance cutoff with different values of target genome completeness fraction.

## DISCUSSION

**FIG 8:**
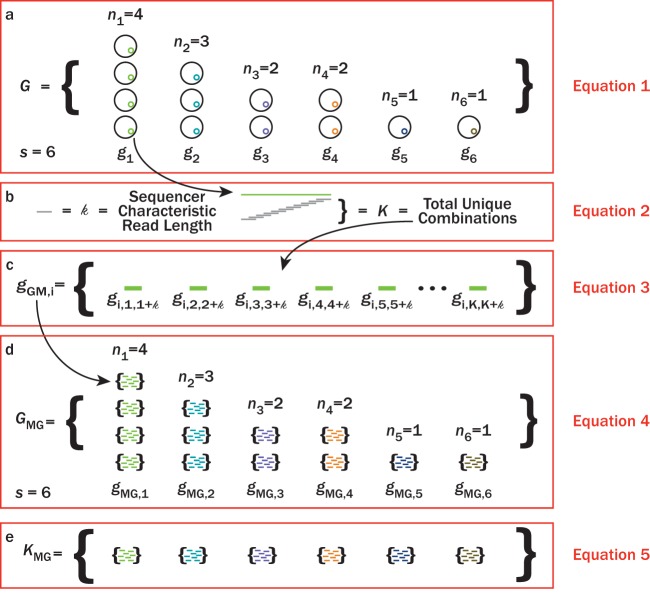
Cartoons illustrating an example microbial community (*G*), the metagenomes for genomes (*g*_MG,i_) (as defined in equation 2) within *G*, and the overall metagenome for the given microbial community (*G*_MG_). In this example, there are six genomes (*s *=* *6) and a total of 13 individual microbes. (a) Black circles represent individual microbes whose genomes are averaged together, *g*. The average genome values, *g*, are indicated by different color inner circles. (b) Individual average genomes can be sequenced at *K* unique positions depending on the characteristic read length, *k*, of a sequencer. (c) All unique positions that can be sequenced for a given genome, *g*, defines the metagenome, *g*_MG_, for the *i*th genome, *g_i_*. (d) Replacing all individual genomes in panel a with metagenomes, *g*_MG_, gives the metagenome of the microbial community, *G*_MG_.

We sought to establish evidence-based guidelines for selecting a sequencing effort during shotgun metagenomic sequencing experiments. The model proposed here (equation 6) addresses this goal. Our model establishes an intrinsic relationship between a community structure and the sequencing effort necessary to sequence members with different rareness, or relative abundance in a community (equation 1; Fig. 8). It is important to emphasize that the proposed model treats individual *k*-mers within a genome as members of the total population of DNA in an environment (equation 4; Fig. 8). Thus, the relative abundance of any given *k*-mer in a population is equal to the abundance of the host genome divided by the total number of *k*-mers in the entire metagenome (equation 7). Summing the probabilities of sequencing all individual *k*-mers from the same genome (equation 2; Fig. 8), should equal the relative abundance of the genome within the population of genomes. The theoretical model utilizes these individual probabilities of sampling *k*-mers to determine how much sequencing effort is required to sequence all possible *k*-mers in a community (Fig. 2 and 3) and for a member of some rareness in a community (Fig. 5). Practical sequencing challenges associated with strain-level microdiversity, extracellular DNA, and sequencer contamination are not problematic for the proposed model. However, these issues can still lead to problems during assembly. In particular, abundant lineages with a large amount of strain-level microdiversity can be entirely missing from the assembled data set despite a high coverage. Strains could be treated as independent taxonomic units, and ultimately, sequencing effort should be selected based on a target rareness of DNA being sequenced in the sequencer. Proper measures such as removing extracellular DNA (e.g., reference 12) and properly removing contaminated DNA are essential to avoid skewing the sequenced DNA population. Last, in practice, homologous DNA regions across genomes (e.g., shared genes) will be sequenced faster than unique regions. For simplicity of our model, we cannot predict the degree of homologous DNA and simply treat unique loci at DNA as a unique *k*-mer. Nonetheless, if this information is known *a priori*, the proposed model can account for homologous DNA. Equation 6 calculates sequencing effort for individual *k*-mer regions based on their relative abundance (*p_j_*) with respect to all *k*-mers in the community. Theoretically, the relative abundances for *k*-mers in homologous regions could be treated as an independent “reservoir,” or genome (*g*_GM,i_; equation 3), such that the relative abundance of the *j*th *k*-mer in this reservoir equals the sum of relative abundances from all host genomes that the homologous DNA actually exists in.

The theoretical probability model (equation 6) demonstrated that the sequencing effort to sequence a metagenome to exhaustion was predictable, regardless of community structure (Fig. 2). Furthermore, our characterization of community complexity demonstrates that less-complex communities require less sequencing to sequence all available DNA (Fig. 4). A limitation to equation 6 is that it predicts only the sequencing effort to sequence a metagenome to completion. In practice, assemblers do not require every unique *k*-mer to generate an accurate contig. As long as sufficient overlap exists between sequenced *k*-mers, assemblers can generate contigs. We therefore used a numerical simulation to predict the sequencing effort necessary to sequence an individual metagenome to a target fraction of exhaustion. The numerical simulation agreed with the theoretical model when predicting the sequencing effort to sequence a metagenome to exhaustion (Fig. 3). A last analysis explored the semiquantitative relationship between community evenness and richness and the necessary sequencing effort to achieve a target fraction of exhaustion for a metagenome (Fig. 4).

In practice, information about a target community structure may not be available for estimating sequencing effort. The GAM built here predicts the minimum number of sequences necessary to sequence a given fraction of a target genome as a function of average genome size and the relative abundance of the target genome in the community (Fig. 5). Even without knowledge of a target community’s structure, the GAM provides a useful constraint for designing whole-genome shotgun metagenomic sequencing experiments. The size of prokaryotic genomes is fairly constrained, with 50% of the genomes in RefSeq v92 spanning from 2.74 Mbp to 5.15 Mbp (11). The limited range in genome size allows for reasonable assumptions about genome size for prokaryotes.

We wanted to relate our model to actual sequencing experiments. As such, the subsampling analysis on existing short-read data sets (individually sampled, assembled, and binned) simulated the effect of creating MAGs from data sets of different sizes from different environments. The data sets analyzed here are representative of both the sequencing effort (1 to 10 Gbp) (5) and the types of target environments that bacterial and archaeal ecologists often investigate (13). We want to emphasize that the data sets analyzed here do not necessarily reflect all the variability in characteristics of their parent communities (i.e., these data sets do not reflect global/temporal variations of these environments). Furthermore, a wide variety of software packages are available for all steps of MAG creation pipelines, and the quantity/quality of MAGs will depend on software selection, software configuration, and sequenced environment (Fig. 1) (5). The best practice is to manually curate algorithmically created MAG bins (14). Due to the large number of metagenomes that were assembled in this work (*n *=* *108), manual curation was an impractical approach. As with most sequencing experiments, viral/eukaryotic DNA is likely included during assembly and binning. Thus, we do not argue that the pipeline used here is objectively optimal for generating high-quality (high-completeness and low-contamination) MAGs. Rather, our pipeline was configured to minimize contamination (MAGs with <10%) associated with retrieved MAGs at the expense of reduced completeness (see Text S1 in the supplemental material). For this reason, we reported the number of quality MAGs (medium- and high-quality MAGs) rather than the actual number of MAGs. Reporting quality MAGs reduces bias associated with the generalized approach used with the binning software. As such, we interpret the data sets analyzed here as reflecting general community properties (richness, abundances, and phylogeny) which are generally known to be different from one another (4, 15–17).

10.1128/mSystems.00384-19.1 TEXT S1 An extended methods section covering sequence data sources, MAG assembly pipeline, relating MAG response to the theoretical sequencing model, subsampling sequence read data sets, and GAM regression. Download Text S1, DOCX file, 0.02 MB. Copyright © 2019 Royalty and Steen. 2019 Royalty and Steen This content is distributed under the terms of the Creative Commons Attribution 4.0 International license.

All communities demonstrated similar responses to rarefaction. All subsampled depths were performed in triplicate to account for possible variation in assembly and binning. As sequencing effort increased, there was an initial lag in quality MAGs followed by a rapid increase in quality MAGs, and then diminishing returns at higher sequencing efforts. Previous investigators modeled the response of 16S RNA gene (18–20), Hill’s number diversity (21), taxon-resolved abundance (22), and gene abundance (22) as a function of sequencing effort with rarefaction curves, or collector’s curves. The quality MAGs as a function of sequencing effort did not match a traditional collector’s curve, which lacks an initial lag. Rather, the data appear sigmoidal. We modeled the data using the Gompertz function (equation 12), because its parameters can be interpreted in terms of quantities that are familiar from microbial growth curves (lag time, growth rate, and maximum density) (10). The fits to the Gompertz function illustrate that there is an optimal sequencing effort for MAG creation efforts corresponding to the upper shoulder of the Gompertz curve (“late log phase”). When sequencing effort is too close to λ, MAGs bin poorly, and when sequencing effort is too great, the number and quality of MAGs per unit sequencing effort (and therefore cost) are low. We speculate that our choice of pipeline, and specifically the fact that we discarded contigs of <3 kb, caused poor performance at higher sequencing effort for the *Tara Oceans* data set. Species-level microdiversity and interspecies homologous DNA can cause “bubbles,” which impair assembly in larger data sets (23, 24). Improved assembly would likely have yielded more quality MAGs for our assembly of the largest subsets of the *Tara Oceans* data.

Metagenomic shotgun sequencing experimental designs should be rationally designed such that sequencing effort is selected to capture a desired fraction of a target microbial genome. Investigators should be cognizant of the rarest microbial genome desired to be characterized as well as the degree of characterization of that microbial metagenome when designing a sequencing experiment. To that end, we have built a tool, Genome Relative Abundance to Sequencing Effort (GRASE), to report estimated sequencing effort required to capture a defined fraction of a genome as a function of the relative abundance of the corresponding microorganism in the community and average genome size. This R-based graphical user interface (GUI) app can be accessed online at http://adsteen.shinyapps.io/grase and is archived at http://github.com/adsteen/GRASE, from which it can be downloaded and run locally.

When the sequence read data sets analyzed here (Table 2 and Fig. 6) are reevaluated in the context of the relative abundance of a microbial metagenome (*g*_MG_) sequenced to a target fraction of exhaustion (0.5), quality MAGs increase appreciably in response to minor increases in deeper characterization of the community metagenome (Fig. 7). This observation contrasts the quality MAGs response to sequencing effort (in base pairs), where substantial increases in sequencing effort (by contemporary standards) leads to diminishing returns in quality MAGs. It is important to note that abundance cutoff and sequencing effort are interchangeable; however, the responses of quality MAGs to changes in the respective predictor (i.e., base pairs versus abundance cutoff) alter the optics of the collector’s curve. Modest increases in sequencing effort contribute minor amounts to extending abundance cutoff. A substantial amount of genomic and metabolic data can be gained from targeting rarer microbes (metagenomic abundances of <0.005), with the caveat that whole-genome shotgun sequencing technology (as well as computational power) requires significant increases in either the number or length of reads generated per run.

**TABLE 2:** Summary of sequence data sets analyzed with the MAG pipeline^*a*^

Environment	NCBI SRA accession no.	Total no. of reads^*b*^	No. of high- quality bases^*b*^	General notes^*c*^	Reference
Ocean surface water	ERR599029	3.372 × 10^8^	3.340 × 10^10^	Caribbean Sea (5 mbsl)	15
Estuary sediment	SRR5248164	1.137 × 10^8^	1.589 × 10^10^	Sulfate zone (8-10 cmbsf)	4
Maize soil	SRR351473	4.727 × 10^8^	3.824 × 10^10^	Surface soil	16
Human gut	SRR5127631	5.095 × 10^8^	4.847 × 10^9^		17

a The sequencing platform used for sequence data sets from all the environments shown was Illumina HiSeq 2000.

b Combined forward and reverse paired-end reads.

c mbsl, meters below sea level; cmbsf, centimeters below sea floor.

## MATERIALS AND METHODS

### Defining the microbial metagenome and sequencing probability.

Here, we draw on set theory to provide a theoretical grounding for our *in silico* simulations described below. The expected number of sequences to sequence a fraction of an individual microbe’s genome can be modeled with probability theory by defining a community metagenome with set theory. Figure 8a to e provide cartoons illustrating the application of this set theory on a hypothetical microbial population, *G*. *G* contains unique genomes (*g*) with finite abundances (*n*). The definition of microbial species is somewhat contentious (25). Here, we define *g* as a genome that is unique in length and composition for all loci compared to all other genomes in a community. As such, the species richness (*s*; unique *g*) of *G* will vary on how it is defined and should be consistent with the objectives of the investigator. In the example communities (Fig. 8a to e), *s *is* *6 and the total *n *is* *13. Thus, *G* is represented as follows (Fig. 8a):

(1) $$G=\left\{n_{1}g_{1},n_{2}g_{2}\ldots n_{s}g_{s}\text{|}n\in N\right\}$$

where *s* is the species richness. When characterizing *G* via shotgun metagenomics, the *i*th genome, *g*_i_, can be sequenced at *K* unique sections given a characteristic read length, *k*, and average genome size, *l*, in number of base pairs (Fig. 8b). Thus, the number of possible *k*-mers, *K*, associated with the *i*th genome, *g*_i_, within *G* is equal to:

(2) $$K_{g_{i}}=l\left(g_{i}\right)-k+1$$

Note that equation 2 considers homologous DNA as unique *k*-mers. This is for model simplification. From equation 2, the metagenome, *g*_MG_, for *g*_i_ is defined as the set of all *k*-mers (Fig. 8c) or:

(3) $$g_{\text{MG},i}=\left\{g_{i,1,1+k},g_{i,2,2+k}\ldots ,g_{i,Kg_{i},Kg_{i}+k}\right\}$$

where the subscripts for *g*_i_ represent a given *k*-sized read spanning from an arbitrary starting base pair to the arbitrary starting base pair plus *k*. By substituting *g*_MG,_*_i_* into all *g* for equation 1 (Fig. 8d), the metagenome for a microbial community, *G*_MG_, is derived to be:

(4) $$G_{\text{MG}}=\left\{n_{1}g_{\text{MG},1},n_{2}g_{\text{MG},2}\ldots n_{s}g_{\text{MG},s}\text{|}n\in N\right\}$$

while the population of *k*-mers in the metagenome, *G*_MG_ (Fig. 8e), is represented as:

(5) $$K_{\text{MG}}=\left\{g_{\text{MG},1},g_{\text{MG},2}\ldots g_{\text{MG},s}\right\}$$

From equation 5, one can determine the cardinality, or the total number, of *k*-mers associated with *G*_MG_ (expressed as |*K*_MG_|). To an effect, |*K*_MG_| is analogous with “metagenomic richness” of an environment. When attempting to fully sequence *G*_MG_ using shotgun metagenomics, we assume that sampling events (sequence reads) are independent and are sampled with replacement (26).

The probability of sequencing all elements in *G*_MG_ reduces to a coupon collector problem (27) by making the above assumptions. Using the general functional form for calculating expected samples for sampling all unique elements in a set (equation 13b in reference 8), one can predict the number of sequences necessary to sequence all elements in *K*_MG_, such that the expected number of sequences, *E*(*G*_MG_), is:

(6) $$E\left(G_{\text{MG}}\right)=\overset{\infty }{\underset{0}{\int }}\left(1-\underset{j\in K_{\text{MG}}}{\prod }\left(1-e^{-p_{j}t}\right)\right)dt$$

where *j* is a given element within *K*_MG_, *t* is the number of sampling events, and *p_j_* is equal to the proportion of the *j*th *k*-sized read within a given population of *k*-sized reads. *p*_j_ can be expressed as follows:

(7) $$p_{j}=\frac{n_{i}\times j\in K_{\text{MG}}}{\left|G_{\text{MG}}\right|}$$

where *n_i_* is the respective abundance for the species whose MAG contains the *j*th *k*-sized read within *K*_MG_, and |*G*_MG_| is the cardinality of *G*_MG_, or the total number of *k*-sized reads in the metagenome, *G*_MG_.

### Modeling expected sequences.

Equation 6 provides an estimate for the total number of sequences to sequence all *K*_MG_. The influence of increasing species richness (i.e., *s* in equation 1) on the expected number of sequences was tested for four hypothetical communities. The first community had an even structure such that all *k*-mers were equally distributed across all *K*_MG_. In the second community, 90% of the *k*-mers were equally distributed in 50% of *K*_MG_, and the remaining 10% of the *k*-mers were distributed equally across the remaining 50% of *K*_MG_. This community represented a community with relatively moderate species evenness. In the third community, 90% of the *k*-mers were equally distributed across 10% of *K*_MG_, and the remaining 10% of the *k*-mers were distributed equally across the remaining 90% of *K*_MG_. This community represented a community with relatively low species evenness. The last community had 10 equally sized groups. The abundance of the *k*-mers in each group was based on the function form of a lognormal community (28) which has been observed in microbial populations (e.g., references 21 and 29), such that:

(8) $$S\left(R\right)=S_{0}e^{-a^{2}R^{2}}$$

where *S*_0_ was treated as the maximum relative of abundance (*S*_0_ = 1), *a* was the inverse width of the distribution, *R* was treated as the positive octave range spanning 0 to 9, and *S*(*R*) represented the abundance for a given group. For the lognormal abundance distribution in Fig. 2d, *a* was set at a value of 0.2. Each hypothetical community started with |*K*_MG_| = 1 × 10^6^. |*K*_MG_| incrementally increased at 10 equally spaced, linear steps to a maximum of |*K*_MG_| = 1 × 10^8^. As |*K*_MG_| increased, all community structures remained constant. Graphical representation of rank abundance in Fig. 2a to d was normalized by a given |*K*_MG_| to reflect that populations retained the same structure even as population size varied. We defined a normalized rank abundance *r_n_* such that

(9) $$r_{n}=\frac{r}{s}$$

where *r* and *s* are untransformed rank abundance and richness, respectively. For each community, at each step, the expected number of sequences was calculated using equation 6. The expected number of sequences as a function of |*K*_MG_| were modeled with linear regressions.

Equation 6 gives the expected number of sequences required to sequence any size community to exhaustion. Numerical sequencing simulations were performed to determine the number of sequences necessary to sequence a subset of all *k*-mers (*K*_MG_). These numerical sequencing simulations were applied to four hypothetical community structures described above. Numerical simulations were performed such that |*K*_MG_| = 3 × 10^7^, 4 × 10^7^, 5 × 10^7^, 7 × 10^7^, 9 × 10^7^, and 1 × 10^8^. During each of these simulations, the parameters read length (*k*) and average genome size (*l*) were set at 100 and 1 × 10^6^, respectively, for all *g*. Random elements from *K*_MG_ were selected with replacement to simulate sequencing events. Numerical simulations were performed until the percentage of |*K*_MG_| sequenced was 50%, 70%, 90%, 95%, 99%, or 100%. A weight distribution was applied to elements in a given *K*_MG_. The weight distribution biased sequencing to reflect the relative abundances of the four hypothetical communities described above. The percentage of |*K*_MG_| sequenced was evaluated every 1 × 10^7^ sequences. Numerical simulations were performed in triplicate for all |*K*_MG_| and all target fractions of |*K*_MG_|.

We explored the influence of community evenness on required sequencing effort by performing sequencing simulations on six different lognormally distributed communities. The numerical sequencing simulations followed the simulations described above. The six lognormal communities were modeled such that each community had *S*_0_ = 1, *R = *10, and |*K*_MG_| = 1 × 10^7^. The values of *a* for the six lognormal distributions were as follows: *a *=* *0, *a *=* *0.005, *a *=* *0.008, *a *=* *0.01, *a *=* *0.015, and *a *=* *0.02. Evenness was represented using the Pielou evenness index (9), or the ratio of the Shannon diversity index (30) for an observed community to an even community of equal richness. Shannon diversity was calculated in the context of a metagenomes such that:

(10) $$H_{\text{MG}}=\underset{j\in K_{\text{MG}}}{\sum }-p_{j}\text{log}\left(p_{j}\right)$$

where *p_j_* is the proportion that the *j*th *k*-sized read represents among all *k*-mers in the metagenome. Thus, the Pielou evenness index (9) was calculated such that:

(11) J=HMG'HMG,max

where *J* was the Pielou evenness index, *H*_MG_′ was the metagenome Shannon diversity index, and *H*_MG,max_ represented the metagenome Shannon diversity index when all *p_j_* were equal (*a *=* *0).

Last, numerical simulations were performed to determine the sequencing effort necessary to achieve a target fraction for an individual metagenome (*g*_MG_). Target fractions increased from 0.5 to 1 at 100 linearly spaced intervals. The fraction of the metagenome community (*G*_MG_) that *g*_MG_ represented varied from 1% to 100% in 30 lognormally spaced intervals. The target genome sizes varied such that *l *=* *0.5 × 10^6^, *l *=* *1 × 10^6^, *l *=* *2 × 10^6^, *l *=* *3 × 10^6^, *l *=* *5 × 10^6^, *l *=* *10 × 10^6^, *l *=* *15 × 10^6^, and *l *=* *20 × 10^6^. The sequencing effort for a given combination of target fraction, genome size, and fraction of the metagenome community was modeled using the GAM function (mgcv R package [31]). Further description of the GAM regression is provided in Text S1 in the supplemental material.

### Sequence data sources.

A more detailed description of the data sources is provided in Text S1. All data sets analyzed in this study are summarized in Table 1.

### MAG assembly pipeline.

The pipeline developed here followed similar pipelines described by other authors (3, 32). A more detailed description of the pipeline is provided in Text S1.

### Subsampling sequence read data sets.

A description of the sampling methodology is provided in Text S1.

### Modeling MAG response to sequencing effort.

Medium-quality and high-quality MAGs were defined on the basis of completeness and contamination from CheckM (33). Medium-quality MAGs were defined as MAGs with >50% completeness and <10% contamination. High-quality MAGs were defined as MAGs with >90% completeness and <5% contamination (14). The sum of medium- and high-quality MAGs as a function of sequencing effort was modeled for environmental sequence data sets using the Gompertz equation, as reformulated by Zweitering et al. (10) for use with microbial growth curves:

(12) $$g_{\text{eq}}\left(A,\text{μ},\text{λ},b\right)=A\times e^{-e^{\frac{\mu \times e}{A}\left(\text{λ}-b\right)+1}}$$

where *A*, μ, and λ are fit coefficients and *b* is high-quality bases (Gbp). MAG yield could be defined as:

(13) $$\text{MAG yield}=\frac{n_{\text{MQ}}+n_{\text{HQ}}}{A}$$

where *n*_MQ_ is the total medium-quality MAGs derived from the subsampling experiment, *n*_HQ_ is the total high-quality MAGs derived from the subsampling experiment, and *A* is from equation 12.

### Relating MAG response to the theoretical sequencing model.

Details of how we related MAG response to the theoretical sequencing model are provided in Text S1.

### Data availability.

All simulations and codes used for modeling sequencing effort are freely available on Github at https://github.com/taylorroyalty/sequence_simulation_code. All data generated during the subsampling experiment is available in Data Set S1 in the supplemental material. The GRASE GUI application is available at http://adsteen.shinyapps.io/grase.
