# Supplementary material for: Theoretical and Simulation-Based Investigation of the Relationship between Sequencing Effort, Microbial Community Richness, and Diversity in Binning Metagenome-Assembled Genomes
Source: mSystems. 2019 Sep 17;4(5):e00384-19. doi: 10.1128/mSystems.00384-19 (PMC6749106; doi:10.1128/mSystems.00384-19)
Supplement: TEXT S1 [file mSystems.00384-19-s0001.docx]

## Sequence Data Sources

All sequence data were downloaded from NCBI's Sequence Read Archive (SRA) using the SRA Toolkit (fastq-dump –split-files) (1). Exact duplicate reads for both forward and reverse reads were removed using PRINSEQ (-derep 1; v0.20.4) (2). All sequencing datasets were limited to Illumina shotgun metagenomic paired-end reads. Four datasets were analyzed for this analysis. The first dataset was from oceanic surface water collected at 5m depth in the Caribbean Sea as a part of the *Tara Oceans* expedition (3). The second dataset was from sediment from a depth of 8-10 cm below the surface (sulfate-rich zone) and collected at the White Oak River Estuary, Station H, North Carolina, USA (4). The third dataset was collected from maize soil (5). The last dataset was collected from human fecal samples and represented a human gut microbiome (6).

## MAG Assembly Pipeline

The pipeline developed here followed similar pipelines described by other authors (7, 8). A more detailed description of the pipeline is provided in the supplementary text. All sequence datasets were analyzed as follows. Trimmomatic (v0.36) (9) removed adapters and trimmed low-quality bases from individual reads. Read leading and trailing quality scores were required to be >3. The sliding window was set to 4 base pairs and filtered base pair windows with a mean score <15. Quality controlled reads were assembled into contigs using MEGAHIT (v1.1.2; --presets meta-large) (10). Due to computational limitations, assembled contigs <3000 bp long were excluded from the analysis. Redundant contigs were removed using CD-HIT (v4.6.8; cd-hi-est -c 0.99 -n 10) (11). The quality-controlled reads (i.e., after using Trimmomatic) were mapped to the remaining contigs using Bowtie 2 (v2.3.3) (12) to generate a coverage score for individual contigs.

Resultant contigs were iteratively clustered into MAGs using the unsupervised, clustering algorithm Binsanity (v0.2.6) (7). Similar to Tully et al. (8), six initial clustering iterations were performed with the parameter, *preference* (-p), set to -10 (iteration 1), -5 (iteration 2), -3 (iteration 3-6). Between iterations, a refinement step (Binsanity-refine) was performed on the putative MAGs with constant *preference* (-p) of -25. The refined putative MAGs were evaluated for contamination and completeness using the software CheckM (v1.0.6) (13), which uses HMMER (v3.1) and Prodigal (v2.6.3) (14). For this work, we used the recommended lineage-specific marker sets. The lineage-specific workflow defines marker genes as those appearing in >97% of a single lineage. Contigs associated with putative MAGs meeting one of the following criteria: 1) had a completeness > 90% and contamination < 10%, 2) had a completeness > 80% and contamination < 5%, or 3) had a completeness > 50% and contamination < 5% were treated as high-quality. All other MAGs were considered low-quality MAGs. MAGs defined as high-quality were not modified any further. Contigs associated with the high-quality MAGs were not used in the subsequent reclustering and refinement steps. The contigs associated with low-quality MAGs were pooled together and reclustered during the next iteration of Binsanity clustering. After the sixth iteration, the remaining MAGs which did not fall into one of the three categories underwent additional refinement using Binsanity-refine. During this step, MAGs were iteratively refined with *preference* set to -10 (iteration 1), -3 (iteration 2), and -1 (iteration 3). Between each refinement step, metrics of contamination and completeness were evaluated using CheckM. Again, MAGs meeting the criteria of a high-quality category described above were not further modified. The respective contigs associated with putative MAGs were not used in proceeding refinement steps. After the last iteration of refinement, all MAGs were reevaluated for completeness, contamination, and a taxonomic rank using CheckM.

## Relating MAG Response to the Theoretical Sequencing Model

Sequencing effort (in base pairs) used for predicting complete-MAG equivalents for the sequence read datasets maize soil, an estuarine sediment, the surface ocean, and the human gut, were related to a genome relative abundance utilizing the GAM presented in the previous section. This was accomplished by setting a constant genome size and target fraction (0.5) and performing a linear regression between genome relative abundance (a genome’s fraction of a community) and log-transformed base pairs. The linear regression was performed with genome sizes of 1 Mbp, 5 Mbp, and 20 Mbp. Complete-MAG equivalents were predicted as a function of genome relative abundance sequenced to a target fraction of 0.5.

## Subsampling Sequence Read Datasets

The effect of decreased sequencing effort was simulated by subsampling the initial sequence read datasets described above. Downloaded sequence read datasets were randomly sampled at set fractions of 1%, 10%, 20%, 40%, 60%, 80%, 90%, 95%, and 100%. Each fraction was resampled, assembled, and binned in triplicate. Each triplicate assembly was binned independently using the MAG assembly pipeline described above.

## Gam Regression

For modeling purposes, target fraction was raised to the 6^th^ power and both genome size and sequences were log-transformed. The number of smooth dimensions for fraction of community, genome size, and target fraction were heuristically varied till the resulting fit demonstrated residuals with a normal distribution. Note that the objective here was not build a predictive model but simply a first order approximation for simulations performed here. The GAM was extrapolated for genome relative abundances spanning 0.01 to 0.0001 at all sizes and target fractions. The extrapolation was necessary due to the computational limitation of analyzing genome relative abundances <0.01.

References

1. Leinonen R, Sugawara H, Shumway M. 2010. The Sequence Read Archive. Nucleic Acids Res 39:2010–2012.

2. Schmieder R, Edwards R. 2011. Quality control and preprocessing of metagenomic datasets. Bioinformatics 27:863–864.

3. Karsenti E, Acinas SG, Bork P, Bowler C, de Vargas C, Raes J, Sullivan M, Arendt D, Benzoni F, Claverie JM, Follows M, Gorsky G, Hingamp P, Iudicone D, Jaillon O, Kandels-Lewis S, Krzic U, Not F, Ogata H, Pesant S, Reynaud EG, Sardet C, Sieracki ME, Speich S, Velayoudon D, Weissenbach J, Wincker P. 2011. A holistic approach to marine Eco-systems biology. PLoS Biol 9:7–11.

4. Baker BJ, Lazar CS, Teske AP, Dick GJ. 2015. Genomic resolution of linkages in carbon, nitrogen, and sulfur cycling among widespread estuary sediment bacteria. Microbiome 3:14.

5. Howe AC, Jansson JK, Malfatti SA, Tringe SG, Tiedje JM, Brown CT. 2014. Tackling soil diversity with the assembly of large, complex metagenomes. Proc Natl Acad Sci 111:4904–4909.

6. Schirmer M, Smeekens SP, Vlamakis H, Jaeger M, Oosting M, Franzosa EA, Jansen T, Jacobs L, Bonder MJ, Kurilshikov A, Fu J, Joosten LAB, Zhernakova A, Huttenhower C, Wijmenga C, Netea MG, Xavier RJ. 2016. Linking the Human Gut Microbiome to Inflammatory Cytokine Production Capacity. Cell 167:1125–1136.e8.

7. Graham ED, Heidelberg JF, Tully BJ. 2017. BinSanity: unsupervised clustering of environmental microbial assemblies using coverage and affinity propagation. PeerJ 5:e3035.

8. Tully BJ, Graham ED, Heidelberg JF. 2018. The reconstruction of 2,631 draft metagenome-assembled genomes from the global oceans. Sci Data 5:1–8.

9. Bolger AM, Lohse M, Usadel B. 2014. Trimmomatic: a flexible trimmer for Illumina sequence data. Bioinformatics 30:2114–20.

10. Li D, Luo R, Liu C-M, Ting H-F, Sadakane K, Yamashita H, Lam T-W. 2016. MEGAHIT v1.0: A fast and scalable metagenome assembler driven by advanced methodologies and community practices. Methods 102:3–11.

11. Fu L, Niu B, Zhu Z, Wu S, Li W. 2012. CD-HIT: accelerated for clustering the next-generation sequencing data. Bioinformatics 28:3150–3152.

12. Langmead B, Salzberg SL. 2012. Fast gapped-read alignment with Bowtie 2. Nat Methods 9:357–9.

13. Parks DH, Imelfort M, Skennerton CT, Hugenholtz P, Tyson GW. 2015. CheckM: assessing the quality of microbial genomes recovered from isolates, single cells, and metagenomes. Genome Res 25:1043–55.

14. Hyatt D, Chen G-L, LoCascio PF, Land ML, Larimer FW, Hauser LJ. 2010. Prodigal: prokaryotic gene recognition and translation initiation site identification. BMC Bioinformatics 11:119.
